# Ezetimibe Improves Rosuvastatin Effects on Inflammation and Vascular Endothelial Function in Acute Coronary Syndrome Patients Undergoing PCI

**DOI:** 10.1155/2021/2995602

**Published:** 2021-09-07

**Authors:** Changqing Sun, Wuyang Zheng, Ling Liang, Zuheng Liu, Wenchao Sun, Rong Tang

**Affiliations:** Department of Cardiology, The First Affiliated Hospital of Xiamen University, Xiamen 361003, China

## Abstract

**Background:**

Little is known of the acute effects of ezetimibe in patients with acute coronary syndrome (ACS) undergoing PCI. We investigated whether ezetimibe improves inflammation and vascular endothelial function in patients with ACS undergoing PCI.

**Methods:**

We randomized 171 patients with ACS undergoing PCI to receive ezetimibe 10 mg/day plus rosuvastatin 20 mg/day (combination group, *n* = 81) versus rosuvastatin 20 mg/day (rosuvastatin group, *n* = 90). Lipid profile, type II secretory phospholipase A2 (sPLA2-IIa), interleukin-1*β* (IL-1*β*), vascular cell adhesion molecule-1 (VCAM-1), and intercellular cell adhesion molecule-1 (ICAM-1) were measured at baseline and after 7 days. Three months after PCI, clinical outcomes were examined.

**Result:**

The levels of sPLA2-IIa and IL-1*β* reduced significantly in both groups, but more when ezetimibe and rosuvastatin were coadministered (sPLA2-IIa: 6.16 ± 2.67 vs. 7.42 ± 3.53 ng/ml, *p*=0.01; IL-1*β*: 37.39 ± 26.25 vs. 48.98 ± 32.26 pg/ml, *p*=0.01). A significant rise of VCAM-1 and ICAM-1 was observed on day 7 after PCI in the both groups, but was less in the combination group (VCAM-1: 918.28 ± 235.31 vs. 988.54 ± 194.41 ng/ml, *p*=0.03; ICAM-1: 213.01 ± 100.15 vs. 246.88 ± 105.71 ng/ml, *p*=0.03). Patients in the combination versus rosuvastatin group appeared to suffer from less major adverse events. Periprocedural therapy of ezetimibe improves rosuvastatin effects on proinflammatory responses and endothelial function associated with ACS patients undergoing PCI. This trial is registered with https://clinicaltrials.gov/ct2/show/ChiCTR-IPR-17012219 (Chinese Clinical Trial Registry, http://www.chictr.org.cn on 02/08/2017).

## 1. Introduction

Statins, inhibitors of 3-hydroxy-3-methylglutaryl coenzyme A reductase, are well-established first-line agents for the primary and secondary prevention of atherosclerotic cardiovascular disease [1, 2], mainly due to their powerful effect on lowering low-density lipoprotein cholesterol (LDL-C) levels [3] and anti-inflammatory property [4, 5]. Deposition of LDL-C in the arterial endothelium and the inflammation activated by it play a key role in the pathogenesis of atherosclerosis [6]. Ezetimibe blocks absorption of dietary and biliary cholesterol from the gut by inhibiting its target molecule, Niemann-Pick C1-like 1 (NPC1L1), a cholesterol transporter enriched in the apical membrane of small intestine absorptive enterocytes where it mediates extracellular cholesterol transport across the brush border membrane [7, 8]. Ezetimibe can further decrease LDL-C levels by 6%–25% when coadministered with a statin [9].

A recent study evaluated the effects of dual LDL-C-lowering therapy with ezetimibe-statin in patients with acute coronary syndrome (ACS) on IVUS-derived coronary atherosclerosis and showed stronger coronary plaque regression during a 9–12-month of follow-up, compared with statin monotherapy [10]. In another trial (the IMPROVE-IT trial), combination therapy with ezetimibe plus statin versus statin alone improved clinical outcomes of ACS patients, including cardiovascular death, myocardial infarction, stroke, unstable angina leading to hospitalization and coronary revascularization ≥30 days during a median 6-year follow-up [11]. Additionally, several studies have demonstrated that combination therapy with ezetimibe plus statin for 6–8 weeks reduces inflammation, aortic stiffness, and oxidative stress and improves endothelial function in patients with acute or stable coronary artery disease and rheumatoid arthritis [12–14]. However, the acute effects of coadministration with ezetimibe plus statin in patients with ACS undergoing percutaneous coronary intervention (PCI) remained unknown. Moreover, the potential mechanisms underlying the clinical benefits of ezetimibe and statin coadministration in patients with ACS have not been explored. We hypothesized that the benefits of combination therapy may be partly related to its effects on inflammation and endothelial function in addition to further cholesterol-lowering. This study was designed to evaluate whether periprocedural combination therapy of ezetimibe plus statin improves postprocedural levels of blood parameters of inflammation (sPLA2-IIa and IL-1*β*) and vascular endothelial dysfunction (VCAM-1and ICAM-1) in ACS patients undergoing selective PCI.

## 2. Materials and Methods

### 2.1. Patients

This study is a single-center, prospective, randomized, and controlled trial conducted and reported in accordance with the requirements of the CONSORT statement. Patients who were hospitalized for ACS within the preceding 10 days, with acute myocardial infarction (MI) with or without electrocardiographic ST-segment elevation or high-risk unstable angina (UA), and underwent PCI based on the recommendations of the Chinese Guidelines [15] were eligible for inclusion. Subjects were excluded if having (1) established malignant tumor; (2) infectious or immunological disease; or (3) severe pulmonary, hepatic, or renal diseases. Additionally, participants were excluded if they were taking statins or ezetimibe prior to enrollment into this study. A total of 171 patients were randomly assigned to either rosuvastatin 20 mg/day, or the combination of ezetimibe (10 mg/day) plus rosuvastatin (20 mg/day), in addition to standard ACS therapy. The study was performed according to the Helsinki Declaration and was approved by the Medical Research Ethics Committee of the Southwest Hospital, Army Medical University (approval code no: 2017–38). Written informed consent was obtained from the participant enrolled in this study.

### 2.2. Blood Analyses

Blood samples for measurements of parameters of inflammation and vascular endothelial dysfunction were drawn at admission and on day 7 after PCI and immediately centrifuged at 4°C for 5 min at 3000 ×g. The plasma was collected and stored at −70°C until analysis. Commercially available enzyme-linked immunosorbent assays (ELISA) kits were used to determine plasma levels of sPLA2-IIa (Cayman Chemicals, Ann Arbor, MI, USA), IL-1*β*, VCAM-1, and ICAM-1 (R&D Systems, Minneapolis, MNUSA). All measurements were performed according to the manufacturers' instructions. Each sample was tested in triplicate, and the mean of the triplicate was used for statistical analysis.

### 2.3. Clinical Follow-Up

After a 7-day treatment with rosuvastatin alone or coadministration of ezetimibe and rosuvastatin, all patients received daily 20 mg rosuvastatin for the secondary prevention of cardiovascular events. A clinical follow-up 3 months post-PCI was done by office visits or telephone calls in all participants. The effects of rosuvastatin alone or combination of ezetimibe and rosuvastatin on middle-term clinical outcomes in these patients were documented, including the occurrences of CV death, nonfatal MI, UA requiring hospitalization, coronary revascularization, and nonfatal stroke [11].

### 2.4. Statistical Analyses

Statistical analyses were performed with SPSS 18.0 for Windows (SPSS, Chicago, IL, USA). Continuous variables were presented as means ± standard deviation (SD), and categorical data were expressed as numbers and frequencies. Two-tailed independent *t* tests and Mann–Whitney *U* tests were used to compare parameters between the two groups. Frequency was tested with the chi-squared test. A 2-tailed *p* value <0.05 was considered statistically significant.

## 3. Result

### 3.1. Characteristics of the Study Subjects

A total of 171 patients with ACS were enrolled in this trial. One patient died 8 days after PCI because of interventricular septum rupture and 2 patients had pneumonia 2 days after PCI. One patient had gastrointestinal hemorrhage 24 hours after PCI and received blood transfusion. All 171 patients (90 patients in the rosuvastatin group and 81 patients in the combination group) completed the 7-day trial according to the protocol. Clinical characteristics and medical therapies in the two treatment groups were not significantly different at baseline (Table 1). The two groups were also similar in coronary anatomy, procedural characteristics, diameter, and length of implanted stents (Table 2). Ezetimibe- or statin-induced toxicity, such as persistent transaminase elevation, myopathy, or rhabdomyolysis, was not observed in patients enrolled in this study.

### 3.2. Plasma Levels of Lipids

At baseline, the plasma levels of total cholesterol (TC), LDL-C, high-density lipoprotein cholesterol (HDL-C), and triglyceride (TG) in the two treatment groups were not significantly different. On day 7 after PCI, a greater reduction in plasma levels of TC and LDL-C was observed in the combination group versus statin monotherapy (TC: 14.1% vs. 5.9%, *p*=0.02; LDL-C: 15.6% vs. 6.1%, *p*=0.04). HDL-C and TG levels were not significantly reduced in each group after 7 days of drug treatment post-PCI and showed no statistical difference between the two groups after the treatment (Table 3).

### 3.3. Plasma Levels of Markers for Proinflammatory Responses and Vascular Endothelial Dysfunction

**Figure 1:**
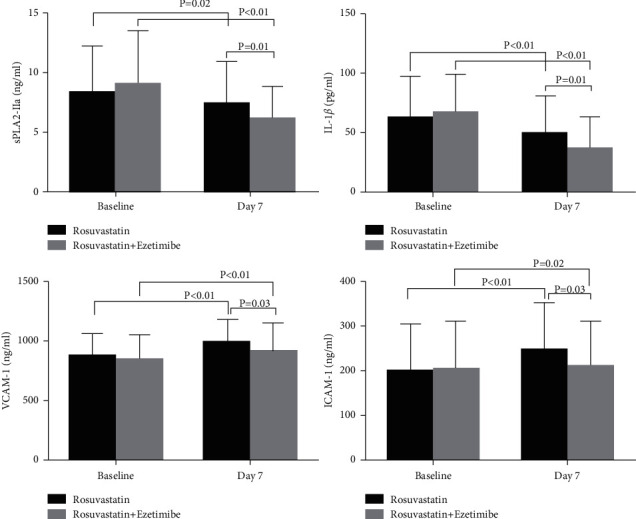
Plasma levels of sPLA2-IIa, IL-1*β*, VCAM-1, and ICAM-1 in patients with ACS on baseline and 7th day after PCI.

Plasma levels of proinflammatory markers sPLA2-IIa and IL-1*β* as well as vascular endothelial dysfunction makers VCAM-1 and ICAM-1 were similar in the two groups at the time of randomization. At the 7th day postprocedure, the plasma levels of sPLA2-IIa reduced significantly from baseline in both groups (rosuvastatin: from 8.32 ± 3.86 ng/ml to 7.42 ± 3.53 ng/ml, *p*=0.02; combination: from 9.11 ± 4.35 ng/ml to 6.16 ± 2.67 ng/ml, *p* < 0.01), and this decrease was significantly lower in the combination versus rosuvastatin group (6.16 ± 2.67 vs. 7.42 ± 3.53 ng/ml, *p*=0.01). Similarly, IL-1*β* levels decreased significantly at the 7^th^ day after the procedure in both groups (rosuvastatin: from 62.99 ± 35.01 pg/ml to 48.98 ± 32.26 pg/ml, *p* < 0.01; combination: from 67.95 ± 31.56 pg/ml to 37.39 ± 26.25 pg/ml, *p* < 0.01). Coadministration of ezetimibe with rosuvastatin resulted in a significant lower level of IL-1*β* than rosuvastatin alone (37.39 ± 26.25 vs. 48.98 ± 32.26 pg/ml, *p*=0.01) (Figure 1).

Unlike plasma sPLA2-IIa and IL-1*β* levels, a significant rise in plasma VCAM-1 and ICAM-1 levels was observed at the 7th day after PCI in the rosuvastatin group (VCAM-1: from 876.32 ± 184.78 ng/ml to 988.54 ± 194.41 ng/ml, *p* < 0.01; ICAM-1: from 201.65 ± 104.04 ng/ml to 246.88 ± 105.71 ng/ml, *p* < 0.01) and in the combination group (VCAM-1: from 843.31 ± 211.87 ng/ml to 918.28 ± 235.31 ng/ml, *p* < 0.01; ICAM-1: from 204.64 ± 107.22 ng/ml to 213.01 ± 100.15 ng/ml, *p*=0.02). However, combination therapy of rosuvastatin and ezetimibe led to significantly lower levels of VCAM-1 (918.28 ± 235.31 vs. 988.54 ± 194.41 ng/ml, *p*=0.03) and ICAM-1 (213.01 ± 100.15 vs. 246.88 ± 105.71 ng/ml, *p*=0.03), compared to rosuvastatin monotherapy (Figure 1).

### 3.4. Clinical Follow-Up Outcomes

To determine whether the 7 days of postprocedure combination therapy of ezetimibe and rosuvastatin has any impact on middle-term clinical outcomes, we followed up the patients for 3 months. The patients in the combination group appeared to suffer from less major adverse events including CV death, nonfatal MI, UA requiring hospitalization, or coronary revascularization during this period, but no statistical difference was observed (Table 4).

## 4. Discussion

This study demonstrates that the coadministration of ezetimibe and rosuvastatin in patients with ACS leads to greater reduction in the proinflammatory factors as reflected in plasma concentrations of IL-1*β* and sPLA2-IIa at the 7th day after PCI, as compared to rosuvastatin treatment alone. The combined therapy versus rosuvastatin alone is better in protecting the coronary angioplasty-induced increase in plasma ICAM-1 and VCAM-1 levels. These findings suggest that combined therapy relative to monotherapy may be a better option for cardiovascular protection in ACS patients undergoing PCI. Additionally, our results show that the 7-day postprocedure coadministration of ezetimibe and rosuvastatin in patients with ACS undergoing PCI improves the major adverse events, including the occurrences of CV death, nonfatal MI, UA, and coronary revascularization, during a three-month follow-up, though no statistical significance is obtained likely due to the short duration of the follow-up. To the best of our knowledge, this trial is the first to document the effects of acute application of aggressive cholesterol-lowering therapy (i.e., ezetimibe plus rosuvastatin) in the periprocedural period of PCI in patients with ACS.

Interleukin 1*β* (IL-1*β*) is a proinflammatory cytokine [16] that has been widely documented to play critical roles in nearly all stages of atherosclerosis from early plaque formation to the destabilization and rupture of advanced lesions [17]. Cholesterol in circulation is taken up by monocyte-derived macrophages [18–20], inducing inflammation by stimulating the caspase-1-activating NLRP3 inflammasome, which results in cleavage and secretion of IL-1*β* and other proinflammatory cytokines [21]. Estruch et al. reported that LDL induces priming and inflammasome activation leading to IL-1*β* release in human monocytes and macrophages [22]. Therefore, IL-1*β* levels may be influenced by lipid-lowering therapy. In our study, ezetimibe/rosuvastatin coadministration relative to rosuvastatin monotherapy achieved a greater reduction in plasma LDL-C and IL-1*β* levels. Similar to our study, Moutzouri et al. [23] reported that high simvastatin dose or the combination of a low-dose simvastatin with ezetimibe reduces IL-1*β* production in monocytes of hypercholesterolemic patients. Another prospective and randomized study in CAD patients demonstrated that atorvastatin markedly downregulates the expression of NLRP3 inflammasome [24], which activates proteolytic enzyme caspase-1 to cleave pro-IL-1*β* producing the active mature IL-1*β* peptide. However, a recent study showed no association between statin use and IL-1*β* levels in a healthy population [25]. The apparent discrepancy among these studies including ours may be explained by the different complications of the subjects and that high-risk subjects versus normal subjects probably benefit more.

In the present study, periprocedural coadministration of ezetimibe and rosuvastatin relative to rosuvastatin alone significantly reduces plasma sPLA2-IIa levels in ACS patients undergoing PCI. It has been shown that rosuvastatin and ezetimibe combination inhibits the cytokines induced by the action of IL-1*β* [26, 27]. Accumulating evidence from basic research and clinical trials has demonstrated that sPLA2-IIa plays an important role in the pathogenesis of atherosclerosis and the instability of the atherosclerotic plaque [28, 29]. Thus, our findings may have important clinical implications.

Recruitment and adhesion of monocytes to the arterial endothelial lining is one of the earliest detectable events during atherogenesis [30]. Endothelial activation molecules (e.g., ICAM-1 and VCAM-1) play crucial roles in the cascade of cell interactions that mediate extravasation and migration of inflammatory cells into the vascular endothelium [31]. Therefore, circulating endothelial activation molecules are regarded as surrogate markers of low-grade vascular inflammation and endothelial dysfunction. Numerous studies have shown that coronary angioplasty is followed by a transient increase in circulating adhesion molecule levels within 24 hours after the procedure [32–34] due to local endothelial damage [34]. Our results confirm these findings by showing that adhesion molecule levels are increased at the 7th day after percutaneous intervention. We did not detect significant correlations between changes in adhesion molecules and LDL-C following lipid-lowering therapy in the two groups, suggesting that improvement in endothelial function may not depend on drugs' lipid-lowering effects. An early study in patients undergoing PCI showed that reduction of procedural myocardial injury after 7-day pretreatment with atorvastatin was paralleled by concomitant attenuation of postprocedural increase of adhesion molecule levels [32]. These findings may explain the protective effects of statin on myocardial damage during coronary intervention observed in the ARMDYDA trial [35]. In a recent trial [36], the benefit of periprocedural loading doses of statins among ACS patients was observed in patients undergoing PCI. Because the reduction of MACE observed in this study occurred early after statin initiation, the mechanism behind this potential effect is likely the statins' pleiotropic effects. However, no previous studies have evaluated the effects of short-term treatment with ezetimibe and a statin on adhesion molecule levels in ACS patients undergoing PCI. Our finding that combined therapy relative to rosuvastatin monotherapy is more effective in attenuating the PCI-stimulated increase in blood adhesion molecules highlighting the possibility that ezetimibe and rosuvastatin coadministration versus rosuvastatin alone may promote better clinical outcomes in patients with ACS undergoing PCI. Indeed, we observed reduced cardiovascular events in the combination group, though our study may be underpowered to obtain statistically significant differences on clinical endpoints between the two groups due to the small sample size and the relatively short observation duration.

## 5. Conclusion

The present study shows that coadministration of ezetimibe and rosuvastatin relative to rosuvastatin monotherapy is more effective in improving plasma markers of proinflammatory responses and vascular endothelial dysfunction in ACS patients undergoing PCI. Our findings may improve clinical practice in these patients. In addition, our observations may help with further clarification of the mechanisms underlying the potential clinical benefits provided by double lipid-lowering therapy, as well as the pharmacological basis of the pleiotropic protection of combination therapy.

## Figures and Tables

**Table 1:** Baseline characteristics of the study subjects.

	Rosuvastatin (*n* = 90)	Ezetimibe/rosuvastatin (*n* = 81)	*p* value
Male, *n* (%)	63 (70.0%)	55 (67.9%)	0.77
Age (years)	64.08 ± 10.45	61.74 ± 8.78	0.12
BMI (kg/m^2^)	24.36 ± 3.18	24.24 ± 3.49	0.82
Current smokers, *n* (%)	44 (48.9%)	34 (42.0%)	0.37
Diabetes mellitus, *n* (%)	30 (33.3)	29 (35.8)	0.74
Hypertension, *n* (%)	50 (55.5)	41 (50.6)	0.52
Dyslipidemia, *n* (%)	37 (41.1)	23 (28.4)	0.08
LVEF (%)	59.00 ± 7.47	60.17 ± 7.87	0.32

Presentation of ACS			
STEMI, *n* (%)	24 (26.7)	16 (19.8)	0.29
NSTEMI, *n* (%)	13 (14.4)	6 (7.4)	0.14
UA, *n* (%)	53 (58.9)	59 (72.8)	0.06

Previous medications			
Aspirin, *n* (%)	10 (11.1)	7 (8.6)	0.59
Clopidogrel, *n* (%)	5 (5.6)	3 (3.7)	0.57
Beta-blockers, *n* (%)	10 (11.1)	5 (6.2)	0.25
ACE inhibitors, *n* (%)	14 (15.6)	9 (11.1)	0.40

Values are presented as *n* (%) or mean ± standard deviation. BMI, body mass index; LVEF, left ventricular ejection fraction; ACS, acute coronary syndrome; STEMI, ST-segment elevation myocardial infarction; NSTEMI, none ST-segment elevation myocardial infarction; UA, unstable angina; ACE, angiotensin-converting enzyme.

**Table 2:** Procedural characteristics.

	Rosuvastatin (*n* = 90)	Ezetimibe/rosuvastatin (*n* = 81)	*p* value
Vessel treated			
Left anterior descending	52 (57.8)	48 (59.3)	0.84
Left circumflex	25 (27.8)	16 (19.8)	0.22
Right coronary artery	33 (36.7)	27 (33.3)	0.65

Type of intervention			
Balloon only	8 (8.9)	5 (6.2)	0.50
Stent	82 (91.1)	76 (93.8)	0.50
No. of stents per patient	1.43 ± 0.63	1.26 ± 0.57	0.10
Stent diameter (mm)	2.98 ± 0.34	3.00 ± 0.34	0.83
Total stent length (mm)	30.37 ± 15.38	26.04 ± 15.29	0.08
Direct stenting, *n* (%)	14 (17.1)	10 (13.2)	0.49
No. of predilatations	68 (82.9)	66 (86.8)	0.49

Values are presented as *n* (%) or mean ± standard deviation.

**Table 3:** Values of blood lipids at baseline and 7 days after PCI.

	Rosuvastatin (*n* = 90)	Ezetimibe/rosuvastatin (*n* = 81)	*p* value
TC (mg/dl)			
Baseline	177.50 ± 53.36	175.95 ± 48.34	0.83
Day 7	167.05 ± 49.50	151.20 ± 37.12	0.02
*p* value	<0.01	<0.01	

LDL-C (mg/dl)			
Baseline	113.69 ± 40.99	114.08 ± 32.87	0.97
Day 7	106.73 ± 37.90	96.29 ± 22.82	0.04
*p* value	<0.01	<0.01	

HDL-C (mg/dl)			
Baseline	39.44 ± 10.44	42.15 ± 10.44	0.13
Day 7	39.06 ± 9.28	40.22 ± 8.89	0.41
*p* value	0.29	0.13	

TG (mg/dl)			
Baseline	183.29 ± 130.16	173.55 ± 122.19	0.47
Day 7	171.78 ± 146.10	168.24 ± 143.44	0.78
*p* value	0.09	0.27	

Values are presented as mean ± standard deviation.

**Table 4:** Clinical follow-up outcomes within the three-month after PCI.

	Rosuvastatin (*n* = 90)	Ezetimibe/rosuvastatin (*n* = 81)	*p* value
CV death, *n* (%)	3 (3.3)	1 (1.2)	0.62
Nonfatal MI, *n* (%)	4 (4.4)	3 (3.7)	0.72
UA, *n* (%)	10 (11.1)	8 (9.9)	0.81
Revascularization, *n* (%)	7 (7.8)	5 (6.2)	0.68
Nonfatal stroke, *n* (%)	0	0	—

Data are summarized as *n* (%). CV, cardiovascular; MI, myocardial infarction; UA, unstable angina.

## Data Availability

The datasets used to support the findings of this study are available from the corresponding author upon request.
